# Postsurgical Prosthetic Rehabilitation after Mandibular Ameloblastoma Resection: A 7-Year Follow-Up Case Report

**DOI:** 10.1155/2021/5593973

**Published:** 2021-03-28

**Authors:** C. Moreno-Soriano, A. Estrugo-Devesa, P. Castañeda-Vega, E. Jané-Salas, J. López-López

**Affiliations:** ^1^Department of Odontostomatology, Faculty of Medicine and Health Sciences (Dentistry), University of Barcelona, L'Hospitalet de Llobregat, Barcelona, Spain; ^2^Oral Health and Masticatory System Group, Institut d'Investigació Biomédica de Bellvitge (IDIBELL, Bellvitge Institute of Biomedical Research), L'Hospitalet de Llobregat, Barcelona, Spain; ^3^Medical-Surgical Area of Dentistry Hospital University of Barcelona-University of Barcelona, L'Hospitalet de Llobregat, Barcelona, Spain

## Abstract

Ameloblastomas are benign but locally invasive odontogenic tumors most frequently located in the mandible. The gold standard of treatment is the surgical resection of the tumor with safety margins. Postsurgical defects generate a significant morbidity that needs reconstruction and oral rehabilitation to restore the oral functions. This case report describes the prosthetic rehabilitation of a 42-year-old male after resection of a mandibular ameloblastoma. Excision of the lesion by segmental mandibulectomy and mandibular reconstruction by microvascularized fibula flap was performed. After placement of 6 dental implants, the patient was rehabilitated with a lower hybrid prosthesis fabricated using computer-aided design-computer-aided manufacturing. During a 7-year and 5-month follow-up, some clinical complications were observed.

## 1. Introduction

Ameloblastomas are benign but locally invasive odontogenic tumors derived from odontogenic epithelium. Their most frequent location is the mandible. No predilection for gender is described and a higher prevalence is shown between the 3^rd^ and 4^th^ decades of life [1]. These lesions represent 1% of oral tumors and 9-11% of all odontogenic tumors [2].

The recurrence rate is 90% if a conservative surgery such as curettage is performed. Otherwise, 15-20% recurrence rates can be achieved with marginal or segmental surgical resection [3].

Ameloblastomas can infiltrate up to 8 mm beyond the apparently healthy margin. Consequently, the gold standard of treatment is surgical resection with ≥1 cm safety margins [3].

Postsurgical defects may generate a significant morbidity that needs reconstruction and rehabilitation techniques to restore the oral functions of patients, increasing their quality of life [4]. Bone reconstruction can be achieved by flaps or bone regeneration materials [5]. Dental implants are the main option for prosthetic rehabilitation. An individualized treatment plan is necessary, which requires multidisciplinary teams: maxillofacial surgeons, implantologists, and prosthodontists.

The aim of the present paper is to present a complex case report of an oral rehabilitation after mandibular ameloblastoma resection, mandibular reconstruction, and placement of six dental implants, with some clinical complications of the peri-implant tissue during the follow-up.

## 2. Case Presentation

A 42-year-old man was referred for prosthetic rehabilitation to the Department of Dentistry for Cancer and Immunocompromised Patients at the Dentistry Hospital of the University of Barcelona.

The patient reported a medical history of controlled arterial hypertension (AHT) and did not have any significant allergies. As toxic habits, he consumed 20 cigarettes daily for 20 years and 1-2 units of alcohol daily.

**Figure 1:**
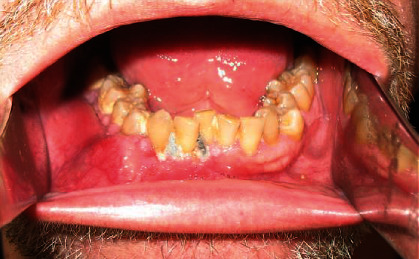
Intraoral image of the lesion. Note the swelling on the right mandibular premolar region.

**Figure 2:**
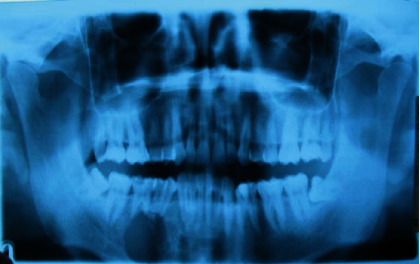
Preoperative orthopantomography.

**Figure 3:**
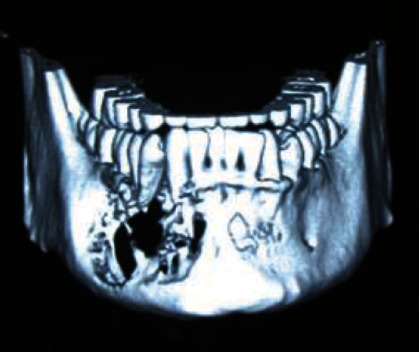
Preoperative computed tomography.

Initially, the patient was referred from his Primary Health Center with an inflammatory process in the right mandible, which was being treated as a dental abscess (Figure 1). Due to nonremission of signs and symptoms, he was referred to the Department of Maxillofacial Surgery of the Head and Neck Unit at Bellvitge Hospital (L'Hospitalet de Llobregat). The following complementary tests were performed: orthopantomography (OPG) (Figure 2), computed tomography (CT) (Figure 3), and biopsy. The final diagnosis was a follicular ameloblastoma.

**Figure 4:**
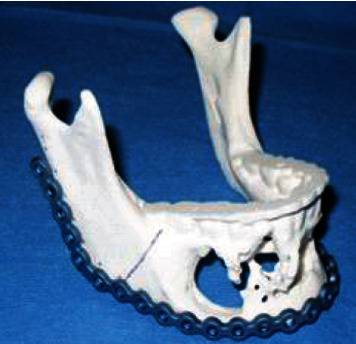
Stereolithographic surgical template from CT and reconstruction plate.

**Figure 5:**
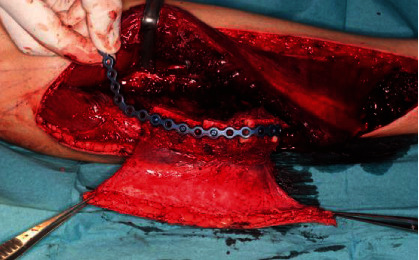
Microvascularized graft of the right fibula and reconstruction plate.

**Figure 6:**
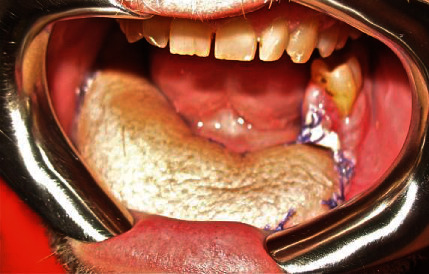
Postoperative intraoral image.

A stereolithographic surgical template from CT scan was created prior to the surgery to design the reconstruction plate and plan the surgery (Figure 4). Excision of the lesion by segmental mandibulectomy of the body and mandibular symphysis of the right side and mandibular reconstruction by microvascularized flap of the right fibula with cutaneous island were performed (Figure 5). A temporary percutaneous tracheostomy was also required. After the first surgery, a 6-month period was allowed to achieve an adequate osseointegration of the flap and soft tissue metaplasia (Figure 6).

**Figure 7:**
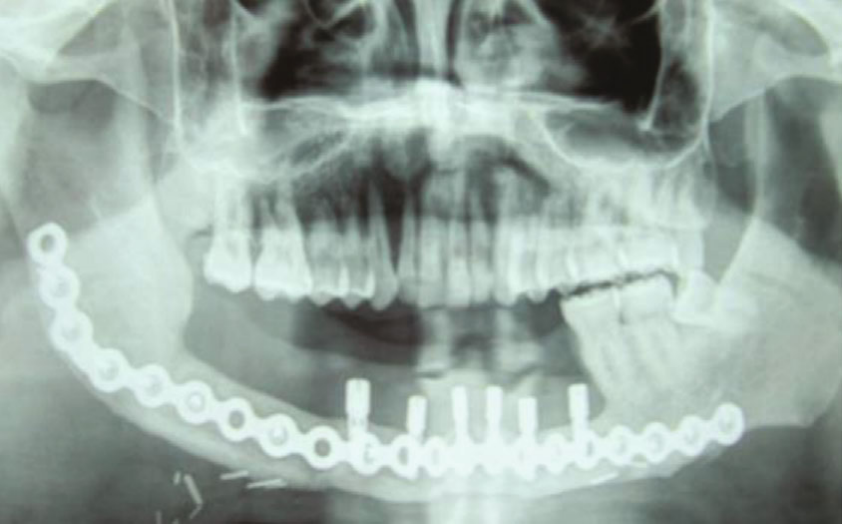
Orthopantomography after 6-implant placement on the fibula.

**Figure 8:**
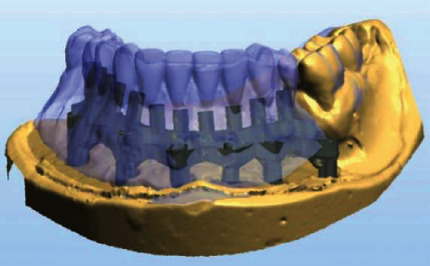
Design of the hybrid prosthesis by CAD-CAM.

**Figure 9:**
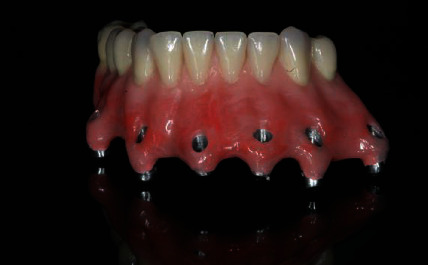
Frontal view of the finished lower hybrid prosthesis.

**Figure 10:**
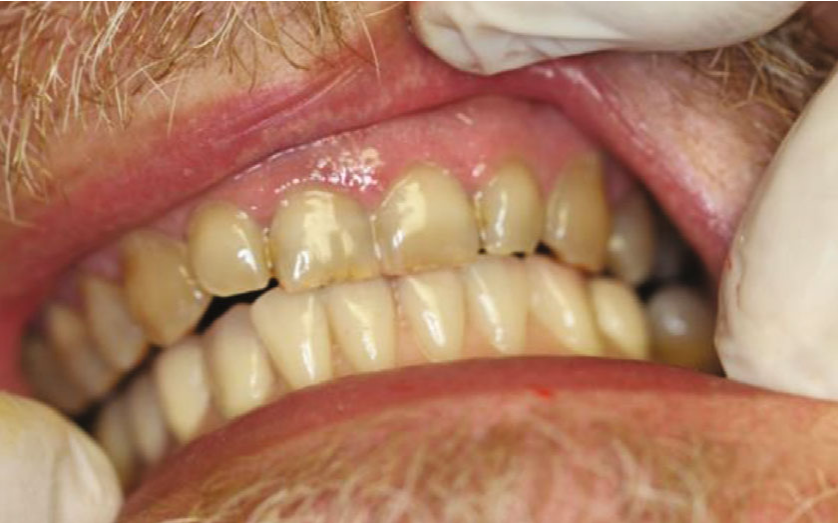
Intraoral image of the hybrid prosthesis placed into the mouth.

Six dental implants (Tapered Screw-Vent; Zimmer Biomet Dental, Warsaw, IN, USA) were placed in the fibula (Figure 7). After the osseointegration of the implants, the patient was referred to the Department of Dentistry for Cancer and Immunocompromised Patients again. After prosthetic planning, the decision was taken to rehabilitate the patient with a lower hybrid prosthesis fabricated by CAD-CAM (Figures 8–10).

Periodic follow-ups were done over a period of 7 years and 5 months. As maintenance program, the hybrid prosthesis was unscrewed and cleaned every six months. Curettage with a plastic curette around the peri-implant tissue and irrigation with chlorhexidine were performed in order to avoid peri-implant disease. In every appointment, oral hygiene instructions were reinforced.

As medical and surgical history, he suffered two myocardial infarctions (MI) and underwent coronary angioplasty with the placement of two stents during the follow-up.

Four years and 7 months after the prosthesis placement, explantation of the most distal implant on the right side had to be done due to a lack of osseointegration (associated with peri-implant disease). Nonetheless, this fact did not affect the stability of the prosthesis.

One year and 5 months later, a biopsy of the peri-implant tissues on the left side was performed to rule out recurrence of ameloblastoma. The histopathological diagnosis was granulation tissue with epithelial hyperplasia without atypical changes.

Six months later, the granulation tissue formation was observed on the right side. As a result, the prosthesis had to be removed. After a month, the tissues showed a clear improvement due to prosthesis removal.

**Figure 11:**
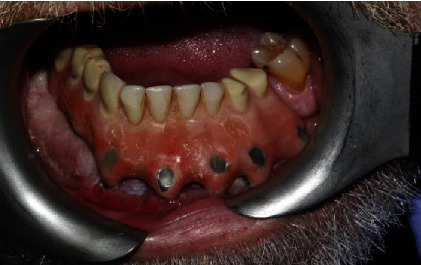
Intraoral image of the patient at the end of the treatment after a 7-year and 5-month follow-up.

Ten months later, as a solution to avoid the recurrence of granulation tissue due to the suspicion of a possible hypersensitivity reaction, the patient underwent partial removal of the metal reconstruction plate. After the healing process, the hybrid prosthesis was placed again and an improvement of the peri-implant tissue was observed after a 7-year and 5-month follow-up (Figure 11).

## 3. Discussion and Conclusion

Because of the high rate of recurrence of ameloblastomas when a conservative surgery is performed, the gold standard of treatment in extensive lesions is tumor resection with ≥1 cm safety margins [5]. Extensive curettages can compromise bone stability. This results in inadequate residual bone for implant placement, which can generate unpredictable results and long periods of oral rehabilitation. On the other hand, a surgery with safety margins ensures complete resection of the tumor, prevents recurrence, and leads to a treatment with a faster and safer rehabilitation [6].

An immediate reconstruction of the postoperative defect is essential to avoid aesthetic problems and functional sequelae such as malocclusions, pathological fractures, or facial asymmetry, leading to concomitant psychosocial problems [5]. The functional reconstruction of large mandibular segments with microvascularized flaps and dental implants is considered the best option. This technique has been widely discussed during the last three decades [7].

Regarding the microvascularized flap, the fibula bone may not provide an adequate height, creating a considerable discrepancy between the reconstructed segment and the occlusal plane [8]. Consequently, implants need longer prosthetic structures in order to approach the occlusal plane, generating excessive leverage forces, overload in the prosthesis, and compromising its long-term success. Moreover, together with a poor oral hygiene, the aforementioned discrepancy between the implants and the occlusal plane creates an ideal space for bacteria growth around peri-implant tissues, which results in granulation tissue growth. To solve this problem, a double-bar fibular flap was proposed [9].

The most remarkable aesthetic and functional benefits are related to implant placement at the same time as the surgical resection of the lesion and mandibular reconstruction. This method allows for placement of implants in the fibula bone structure at a suitable height for a correct and restores occlusion in a single step. Furthermore, this speeds up the oral rehabilitation process and decreases the number of surgeries for the patient [10]. In this case report, implants were placed 6 months after the segmental mandibulectomy, so the patient underwent two different surgeries. As a matter of fact, dental implants were not in a prosthetically driven position because of the anatomy and the bone availability of the fibula, apart from proximity to the reconstruction plate, which compromises the choice of the prosthetic treatment plan.

In reference to oral rehabilitation, a screw-retained hybrid prosthesis was selected as prosthetic treatment due to implants' position with regard to the upper teeth. The possibility of an overdenture was considered, but the corresponding bar or abutments would be too buccally positioned compared to the occlusal plane, which would compromise long-term stability. Moreover, there was a lack of sufficient labial and buccal sulcus to create space for an overdenture flange.

Another important factor is the follow-up period. As a result of the high rate of recurrence of ameloblastoma, it is important to have an exhaustive control of patients. Nevertheless, there is no consensus in the follow-up time related to this type of lesions. Also, the complementary tests play an essential role in these patients. Oral hygiene measures and patient collaboration are vitally important, because clinical complications with the prosthesis may occur, especially food retention, peri-implant disease, and stimulation of the metaplastic tissue of the reconstruction flap. In this case, follow-up was performed over a period of 7 years and 5 months. As maintenance program, the hybrid prosthesis was unscrewed and cleaned every six months. Curettage with a plastic curette around the peri-implant tissue and irrigation with chlorhexidine were performed in order to avoid peri-implant disease. In every appointment, oral hygiene instructions were reinforced.

## Data Availability

The datasets used and/or analyzed during the current study are available from the corresponding author on reasonable request.
